# Supplementary material for: The reimbursement coverage decisions and pricing rules for medical devices in Taiwan
Source: GMS Health Innov Technol. 2022 Mar 16;16:Doc02. doi: 10.3205/hta000134 (PMC8953662; doi:10.3205/hta000134)
Supplement: Table 2: The pricing mechanisms for medical devices in Taiwan, Japan and South Korea [file HInT-16-02-s-001.pdf]

Table 2: The pricing mechanisms for medical devices in Taiwan, Japan and South Korea

Country	Reimbursement pricing mechanism		Price adjustment
	Existing functional category	New functional category (Innovative functional category)	
Taiwan [8], [18]	The lowest reimbursement points in the same existing functional category	**Improved functional category[a]:**<br>1. The lowest international price<br>2. The international price ratio method<br>3. The median, mean, or lowest price of awarded purchase prices adopted by public hospitals in accordance with the Government Procurement Act divided by the average floating-point value for the hospital sector under the hospital global budget as reckoned in the latest four quarters before listing<br>4. The median, mean, or lowest self-pay fee charged by all levels of medical institutions<br>5. The treatment course–expense ratio method<br>6. The reimbursement points for existing medical devices in a similar function category<br>7. The ratio conversion method; the reimbursement points ratio of the existing medical device(s) without the same additional function of the new item to the one(s) with the function shall be applied to the conversion<br>8. The submitted reimbursement points are lower than the above methods, and the submitted reimbursement points could be adopted<br>**Innovative functional category:**<br>1. The price of the original country<br>2. The median of international prices<br>3. The median of awarded purchase prices adopted by public hospitals in accordance with the Government Procurement Act divided by the average floating-point value for the hospital sector under the hospital global budget as reckoned in the latest four quarters before listing<br>4. The median self-pay fee charged by all levels of medical institutions<br>5. Cost calculation method<br>6. The submitted reimbursement points are lower than the above methods, and the submitted reimbursement points could be adopted	Actual transaction price (called Price volume survey)
Japan [2], [10], [21], [29]	**Similar function category comparison method (general rule):**<br>• **No premium**<br>• **Additional premium**<br>Epochal function premium 50% to 100%[b]<br>Utility premium 5% to 30%[c]<br>Improvement premium 1% to 20% (maybe 1% to 10%)[d]<br>Orphan device premium (I): 10%[e]	**Cost accounting system:**<br>1. Production (import) costs<br>2. Sales and general administrative costs<br>3. Operating profits<br>4. Distribution costs<br>5. Consumption taxes	1. Market price<br>2. FAP<br>3. Profitable<br>4. Category restructuring

Attachment to: Tsai HY, Huang YW, Chang SY, Lin CJ, Lee PC, Huang LY. The reimbursement coverage decisions and pricing rules for medical devices in Taiwan. GMS Health Innov Technol. 2022;16:Doc02. DOI: 10.3205/000134, URN: urn:nbn:de:0183-0001348. Available from: https://www.egms.de/en/journals/gms/2022-16/000134.shtm

Country	Reimbursement pricing mechanism		Price adjustment
	Existing functional category	New functional category (Innovative functional category)	
	Orphan device premium (II): 1% to 5%[f]		
South Korea [10], [31], [32], [33]	• **Standard price is the same as existing item**[g]<br>• **Additional improvement premium (comparison - similar items with the same purpose)**<br>VAS (I) adds up to 100%: Based on clinically supporting evidence[h]<br>VAS (II) adds up 50%: Based on the technical supporting evidence[i]	1. Cost accounting calculation method: pricing is a consideration of the existing treatment costs with similar disease states<br>2. Price in other countries<br>3. Manufacturing costs (import FOB)<br>4. Merchandise price	1. Actual transaction price<br>2. Foreign exchange rate

FAP, foreign average pricing; VAS, value appraisal standard; FOB, free on board

[a] If special devices pricing is based on the treatment course–expense ratio method or an existing special device in a similar function category, the following criteria could be considered to yield an additional 15% in reimbursement:

1. Better clinical efficacy
2. Safer for patients or healthcare
3. Improvements in treatment procedures
4. Invasiveness reduction
5. Cost savings
6. Easy for children or operators to use
7. Used by patients with a rare disease or a relatively small group of patients

[b] The new functional Special Designated Treatment Material (STM) that fulfills all of the following requirements:

1. STM has a novel function of clinical utility.
2. STM is objectively shown to have better clinical utility or safety than existing STM.
3. STM is objectively shown to improve the method of treatment for target disease or wounds.

[c] The new functional STM that fulfills any one of requirements for the epochal function premium.

[d] The new functional STM that fulfills any one of the following requirements:

1. STM is safer for the healthcare provider than the existing similarly functioning device.
2. STM has less impact on the environment than the existing similarly functioning device.
3. STM indicates more safety and effectiveness and is less invasive than the existing similarly functioning device.
4. STM has shown that the device is a treatment for infants or children through miniaturization improvements in design.
5. STM enables safer and simpler procedures than existing similarly functioning device.
6. STM indicates that a device is more durable than existing similarly functioning device.
7. STM is shown to be a safer and simpler home treatment for patients than the existing similarly functioning device.

[e] New functional STM is designated as an orphan drug for rare diseases under the Pharmaceutical Affairs Law.

[f] New functional STM has fewer target groups than existing similarly functioning categories.

[g] If a new medical device, it is the same as an existing item (purpose of use, clinical values, appearance, among others).

[h] Including clinical usefulness (therapeutic effects, adverse effects, and improved patient quality of life), cost-effectiveness, and technology innovation.

[i] Including clinical usefulness (functional improvement and procedural easiness), cost-effectiveness, and technology innovation.

Attachment to: Tsai HY, Huang YW, Chang SY, Lin CJ, Lee PC, Huang LY. The reimbursement coverage decisions and pricing rules for medical devices in Taiwan. GMS Health Innov Technol. 2022;16:Doc02. DOI: 10.3205/000134, URN: urn:nbn:de:0183-0001348. Available from: https://www.egms.de/en/journals/gms/2022-16/000134.shtm
